# High-throughput differential screening of mRNAs by serial analysis of gene expression: decreased expression of trefoil factor 3 mRNA in thyroid follicular carcinomas

**DOI:** 10.1038/sj.bjc.6601702

**Published:** 2004-03-09

**Authors:** T Takano, A Miyauchi, H Yoshida, K Kuma, N Amino

**Affiliations:** 1 Department of Laboratory Medicine, Osaka University Graduate School of Medicine, D2, 2-2 Yamadaoka, Suita, Osaka 565-0871, Japan; 2 Kuma Hospital, 8-2-35, Simoyamate-Dori, Chuo-Ku, Kobe, Hyogo 650-0011, Japan

**Keywords:** thyroid, follicular carcinoma, follicular adenoma, serial analysis of gene expression, trefoil factor 3, real-time quantitative RT–PCR

## Abstract

To find mRNAs whose expression differs between thyroid follicular adenomas and carcinomas, a high-throughput analysis of mRNAs in these two tumours was performed. This method, named high-throughput differential screening by serial analysis of gene expression (HDSS), combines a modified method of serial analysis of gene expression (SAGE) and real-time quantitative reverse transcription polymerase chain reaction (RT–PCR). A total of 40 candidate tag sequences that showed extremely different expression levels between a follicular carcinoma and a follicular adenoma in the SAGE analysis were analysed by real-time quantitative RT–PCR, using RNAs from an additional four typical follicular carcinomas and adenomas. One sequence tag that represents trefoil factor 3 (TFF3) mRNA showed a clear difference in its expression level between adenomas and carcinomas. The expression levels of TFF3 mRNA in 48 follicular adenomas and 29 follicular carcinomas were measured by real-time quantitative RT–PCR using a specific probe for TFF3. They were significantly decreased in follicular carcinomas, especially in widely invasive types and those with evident metastases. These results indicate that the decreased expression of TFF3 mRNA is a marker of follicular carcinomas, especially those with a high risk of invasion or metastasis.

Modern advances in molecular technology have given us the chance to establish new strategies in diagnosing cancer. In order to implement such molecular-based technology, it is crucial to identify a distinct difference between benign and malignant cells (Takano and Amino, 2002). Thyroid tumours are relatively common, especially in women. They are often diagnosed by fine needle aspiration biopsy (FNAB) as well as by ultrasonography (Hamburger, 1994; Yokozawa *et al*, 1995). Cytological examination of FNAB by a skillful pathologist who is an expert in thyroid tumours provides the most reliable information for the diagnosis of thyroid neoplasms. In some clinical situations, however, a more objective method is required for exact diagnosis.

In the light of this, we developed a new method of preoperative molecular-based diagnosis of malignant tumours, named aspiration biopsy RNA diagnosis (ABRD), which utilises extracted RNAs from leftover cells within the needle used for FNAB for reverse transcription–polymerase chain reaction (RT–PCR) analysis (Takano *et al*, 1997). For the thyroid, by detecting the specific changes in mRNAs in malignant thyroid tissues by ABRD, an accurate and objective diagnosis can be made in papillary, anaplastic and medullary carcinomas and some malignant lymphomas (Takano *et al*, 1998a, 1999a, 2000a).

Follicular carcinoma of the thyroid is a relatively uncommon malignancy and it accounts for about 15% of all thyroid cancer. One of the most difficult distinctions in thyroid pathology is the differentiation between benign follicular adenoma and the encapsulated low-grade follicular carcinoma called minimally invasive carcinoma. The principal differentiating feature is capsular invasion. Even this, however, may not be definitive, because a slight capsular penetration can also be observed in benign tumours. Further, both types of tumours have varying degrees of cellular atypia, and extensive invasion into vascular spaces is not usually observed in minimally invasive carcinomas. Preoperative diagnosis of follicular carcinoma is even more difficult, since the feature that separated the benign and malignant follicular tumours is the presence of capsular invasion, which is not possible to determine cytologically (Cooper and Schneyer, 1990).

A promising tool for solving this problem is molecular-based diagnosis, such as ABRD. However, at present, ABRD cannot be applied to the preoperative diagnosis of follicular carcinomas because no mRNAs that distinguish adenomas from carcinomas have been reported so far. By relying on 14–15 base cDNA sequences for gene identification, serial analysis of gene expression (SAGE) can generate a quantitative transcript profile easily, a task currently not possible using alternative transcript imaging technologies (Velculescu *et al*, 1995). Since its introduction in 1995, SAGE has been used to analyse cDNA libraries derived from several carcinomas and its reliability has been established. Recently, we established the gene expression profiles of normal thyroids and thyroid tumours and reported some specific changes in mRNAs in thyroid carcinomas (Takano *et al*, 2000b, 2001, 2002a, 2002b; Hasegawa *et al*, 2002). However, even using SAGE analysis, identification of differentially expressed mRNAs in follicular carcinomas was still quite difficult because of the close similarity in the gene expression of follicular adenomas and carcinomas. To solve this problem, it is necessary to perform a high-throughput second screening of candidate sequences, although this is not possible by using the original protocol of SAGE mainly because of the very short sequence tags generated.

A recent modification in the SAGE protocol can construct a gene expression profile with 18-bp tag sequences (Ryo *et al*, 2000). By using this modification, we established a combined analysis of SAGE and real-time quantitative RT–PCR, named high-throughput differential screening by SAGE (HDSS). In this study, by using HDSS, we screened differentially expressed genes in a follicular carcinoma and a follicular adenoma, and found a decreased expression of trefoil factor 3 (TFF3) mRNA in follicular carcinomas. Further, we measured the expression levels of TFF3 in each thyroid malignancy by real-time quantitative RT–PCR.

## MATERIALS AND METHODS

### Extraction of RNA from thyroid tissues

Tissue samples from 19 normal thyroid tissues in the opposite lobe from carcinomas, nine medullary carcinomas, 10 adenomatous goitres, 48 follicular adenomas, 29 follicular carcinomas, 25 papillary carcinomas, and five anaplastic carcinomas were obtained by surgery after patients' informed consent. Tumours were classified according to the WHO histological classification of thyroid tumours by skillful pathologists (Hedinger *et al*, 1989). All tissues were frozen in liquid nitrogen immediately after resection. Total RNA was extracted according to the method of Chomczynski and Sacchi (1987).

### Reverse transcription

Reverse transcription was performed using either 1 *μ*g of total RNA in an RT mixture containing 50 mM Tris-HCl (pH 8.3), 75 mM KCl, 10 mM dithiothreitol, 3 mM MgCl_2_, 0.5 mM dNTPs, 200 U M-MLV reverse transcriptase (Gibco, Gaithersburg, MD, USA), 2 U *μ*l^−1^ RNase inhibitor (Takara, Shiga, Japan), and 2.5 *μ*M oligo dT (Gibco) in a total volume of 20 *μ*l at 37°C for 60 min.

### HDSS procedures

In total, 50 *μ*g of total RNA from a follicular adenoma and a widely invasive follicular carcinoma were used for the construction of gene expression profiles. Modified SAGE was performed as described previously (Ryo *et al*, 2000). Then thousand tags were analysed in each sample. After analysis by PROGENEX software, which was kindly provided by Dr Toru Wakatsuki of the National Defense Medical College (Tokorozawa, Japan), tag sequences for which expression differed greatly between the two tissues were detected. According to these data, 40 18-bp sequences were selected as 5′ primers in the following analysis (Table 1

**Table 1:** Primers used in HDSS

		**Tag count^a^**				
	**Sequence (GTAC–)**	**FC**	**FA**	**Gene bank match**	**Selected 3′primer (CGCGCGT_13_)**	**Result^b^**
F1	CTGCCGCCGCCGCC	0	61	Cellular retinoic acid-binding protein	-GC	B
F2	TCGGGGAGCTGCGG	1	27	No match	-GC	B
F3	TATTTAACTGACTA	0	18	Tumour rejection antigen (gp96) 1 (TRA1)	-CT	C
F4	GTGGGCCTGTCTGC	5	74	Trefoil factor 3	-CG	A
F5	CAGCCGGGCCAGCA	1	13	Cell membrane glycoprotein, 110000M(r)	-GC	B
F6	TCCAGCGCTGGGAG	0	9	No match	-GA	B
F7	CGGGAGAGGCAGGT	0	9	Oxidative 3 alpha hydroxysteroid dehydrogenase	-GC	B
F8	CTTCCCACTGGCCT	0	8	Histone deacetylase 1 (HDAC1)	-CA	B
F9	CGGCCCCCGCCGCC	0	7	cDNA, 3′ end (Hs.205805)	-GC	B
F10	CCAACGGGCCGGGG	0	7	No match	-GC	B
F11	ACTGCAGCCTCCAA	0	7	Interferon, alpha-inducible protein	-GT	B
F12	CTGGCATGGCAGCA	0	6	GSTT1	-GT	C
F13	TGGGTCTGTTGGCC	0	5	SUMO-1 activating enzyme subunit 1 (SAE1)	-GC	B
F14	TGGGCACAGGCCGC	0	5	(Clone hKvBeta3) K+ channel beta subunit	-GA	B
F15	GACGAGTCGGGCCC	0	5	Actin, gamma 1 (ACTG1)	-GG	D
F16	CTTGCGGCCCGTGT	0	5	No match	-GA	B
F17	CTGCGGCCGCCTCG	0	5	cDNA DKFZp566J153	-GC	D
F18	CTCAGAAGTGTGCC	0	5	No match	-GC	D
F19	CGATGCGTGGGCTG	0	5	TM7XN1 protein	-GA	C
F20	CCTGCATCACTGCC	0	5	No match	-GC	C
C1	TTCGCCGAGAGGGT	15	1	Cysteine-rich protein 1 (intestinal) (CRIP1)	-CC	B
C2	CGCCGCCGCGCCGC	12	0	No match	-GG	C
C3	CTGACTTGGGCATC	22	2	SNC73 protein (SNC73)	-GG	B
C4	CTCACGCTTATCAG	10	1	No match	-CA	C
C5	CCCAGACGTCTGGT	10	0	No match	-CC	B
C6	TTTCAACCATCTAC	6	0	No match	-CC	B
C7	CTTTGAACAGCAAC	6	0	Unknown mRNA	-CA	C
C8	CTGCTGCTCAAGCC	6	0	Clone 333H23 on chromosome 22q12.1–12.3.	-GT	B
C9	CGGAAGCGAGAGTG	6	0	EST(BM782041)	-GG	B
C10	CCGGGAGGCGGAGG	6	0	Chromosome 16 BAC clone	-GA	B
C11	CCAGCCCTTGTGTG	6	0	No match	-GG	C
C12	CACCATGCCTGGCT	6	0	cDNA, 3′ end (Hs.155873)	-GC	B
C13	TGGTCTTCGCCTTG	5	0	No match	-CA	B
C14	TCTGCTGAGTGGGC	5	0	Aldehyde dehydrogenase 10 (ALDH10)	-CC	B
C15	TCCAGCCTGGGCTA	5	0	cDNA, 3′ end (Hs.53106)	-GA	C
C16	CGTCGAGGTCAGTC	5	1	cDNA, 3′ end (Hs.30807)	-CA	C
C17	CCCAAGGCCAGGCC	5	0	No match	-GC	B
C18	CCAGACTGCCCACC	5	0	No match	-GC	B
C19	CACCACTCACAGCT	5	0	Aldo-keto reductase family 7, member A2	-CG	B
C20	AGGTGCCCTCTGTG	5	0	Laminin receptor 1 (67 kDa, ribosomal protein SA)	-AG	C

a FC=follicular carcinoma; FA=follicular adenoma.

b A=a positive result in the second screening; B=a negative result in the second screening; C=discordant results in SAGE and RT–PCR; D=no amplification of a single band.

): using one of the 12 primers, CGCGCGT13VN, as the 3′ primer, cDNA from the corresponding tissue, either the follicular adenoma or the follicular carcinoma, was amplified by real-time quantitative PCR. By real-time quantitative PCR analysis with the ABI PRIZM 7700 Sequence Detection System using an SYBR Green PCR Master Mix (Applied Biosystems, Warrington, UK), a 3′ primer with the most optimal amplification was selected. As shown in Figure 1

**Figure 1:**
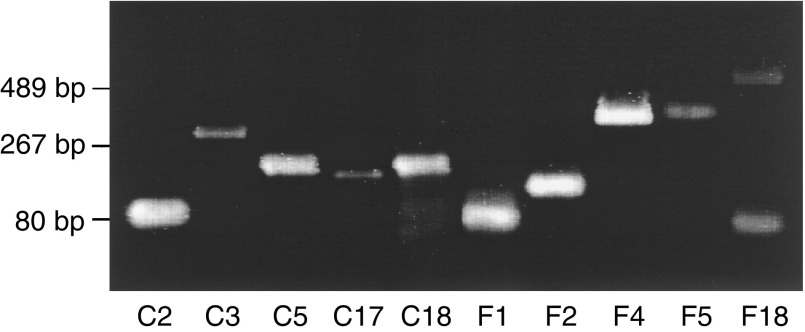
A representative gel image of the PCR products amplified by the primer sets designed for the HDSS analysis. After real-time quantitative RT–PCR analysis using the primer sets described in Table 1, the PCR products were run on a 2% agarose gel. In this figure, all primer sets except F18 amplified a single band.

and Table 1, a single band was observed in the PCR products of 37 of the 40 primer sets after gel electrophoresis. The results of real-time quantitative RT–PCR using cDNA from the two tissues and the data of the SAGE analysis were compared. The differences in expression levels analysed by RT–PCR agreed with the SAGE data in 26 of the 37 sequences. With these 26 primer sets, a second screening by real-time quantitative PCR analysis was performed using cDNAs from four other follicular adenomas and four follicular carcinomas (three widely invasive and one minimally invasive).

### Real-time quantitative PCR

Real-time quantitative PCR (TaqMan PCR) using the ABI PRISM 7700 Sequence Detection System was performed as described previously (Takano *et al*, 1999b). In total, 1 *μ*l of the first-strand cDNA was used in the following assay. The two primers and one TaqMan probe used for the quantification of TFF3 and *β*-actin mRNA (Ponte *et al*, 1984; Hauser *et al*, 1993) were:

TFF3F (0.5 *μ*M): 5′-AATGCACCTTCTGAGGCACCT-3′ (base 265–285); TFF3R (0.5 *μ*M): 5′-CGTTAAGACATCAGGCTCCAGAT-3′ (base 414–436); and TFF3-TM (10 pmol): 5′-FAM-CATCTCAGCTTTTCTGTCCCTTTGCTCCC-TAMRA-3′ (base 359–387); ACF (0.5 *μ*M): 5′-TGGACATCCGCAAAGACCTG-3′ (base 901–920); ACR (0.5 *μ*M): 5′-CCGATCCACACGGAGTACTT-3′ (base1047–1066); and AC-TM (10 pmol): 5′-FAM-CACCACCATGTACCCTGGCATTGCC-TAMRA-3′ (base 947–971), respectively. The conditions for the TaqMan PCR were as follows: 95°C for 10 min and 40 cycles of 95°C for 15 s and 60°C for 1 min. Recombinant pGEM Easy T-Vectors (Promega, Tokyo, Japan) containing either TFF3 or *β*-actin cDNA were constructed by PCR-cloning with the same set of primers used in TaqMan PCR and were used as standard samples.

### *In situ* hybridisation

*In situ* hybridisation was performed essentially as described previously (Takano *et al*, 1998b). In total, 7-*μ*m-thick frozen sections from a follicular adenoma were used in this study. Digoxigenin-labelled single-strand RNA probes were prepared using a DIG RNA labelling kit (Roche, Tokyo, Japan), according to the manufacturer's instructions. For the generation of the sense and antisense probes of the TFF3 sequence, a sequence of human TFF3 cDNA (base 100–359) obtained from a thyroid tissue was subcloned into pGEM Easy plasmid.

### Statistical analysis

Statistical analysis of differences between the groups was carried out using the Mann–Whitney *U*-test. *P*'s of <0.05 were considered significant.

## RESULTS

**Figure 2:**
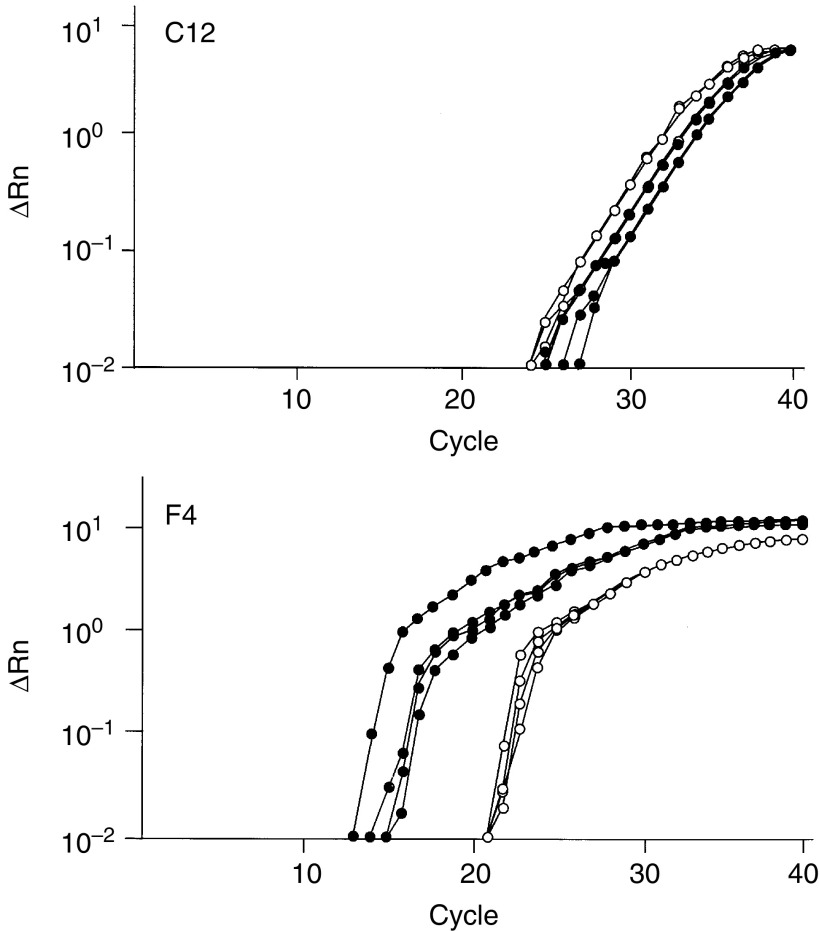
Representative results of the second screening by real-time PCR. cDNAs from four follicular adenomas (closed circles) and four follicular carcinomas (open circles) were amplified by the corresponding primer sets, and the quantity of the PCR products was measured. C12 and F4 showed negative and positive results, respectively.

**Figure 3:**
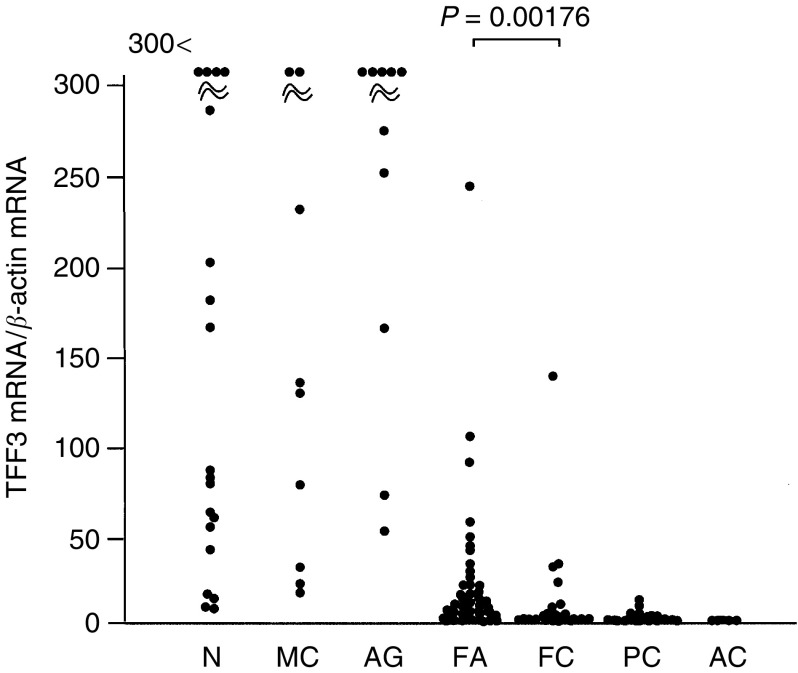
Expression levels of TFF3 mRNA relative to *β*-actin mRNA in thyroid tumours. The results are shown as the mean from duplicate determinations. N, normal thyroid tissues; MC, medullary carcinomas; AG, adenomatous goitres; FA, follicular adenomas; FC, follicular carcinomas; PC, papillary carcinomas; and AC, anaplastic carcinomas.

In the second screening by real-time quantitative RT–PCR, one sequence, F4, showed different expression levels between follicular adenomas and carcinomas but the other 25 sequences did not show clear differences between the two groups (Figure 2

). By sequence analysis, the cDNA amplified by F4 primer was determined to be TFF3. The expression levels of TFF3 mRNA were measured by real-time quantitative RT–PCR using specific primers and a probe for the TFF3 gene and the values were divided by the expression levels of *β*-actin mRNA. TFF3 mRNA was abundantly expressed in normal thyroid tissues, adenomatous goitres, and medullary carcinomas, whereas decreased expression was observed in follicular adenomas, follicular carcinomas, and especially in papillary and anaplastic carcinomas (Figure 3

and Table 2

**Table 2:** Expression of TFF3 mRNA in thyroid tumours

**Tissue**	**Number**	**TFF3 mRNA/*β*-actin mRNA (mean±s.e.)**	**Minimum**	**Maximum**
Normal thyroid	19	210.5±58.6	8.4	829.3
Medullary carcinoma	9	198.8±82.5	16.9	785.0
Adenomatous goitre	10	323.8±60.7	52.6	599.2
Follicular adenoma	48	20.2±5.6	0.019	240.5
Follicular carcinoma	29	9.2±4.8	0.014	135.7
Papillary carcinoma	25	2.0±0.5	0.002	11.3
Anaplastic carcinoma	5	0.2±0.2	0.002	1.1

).

**Figure 4:**
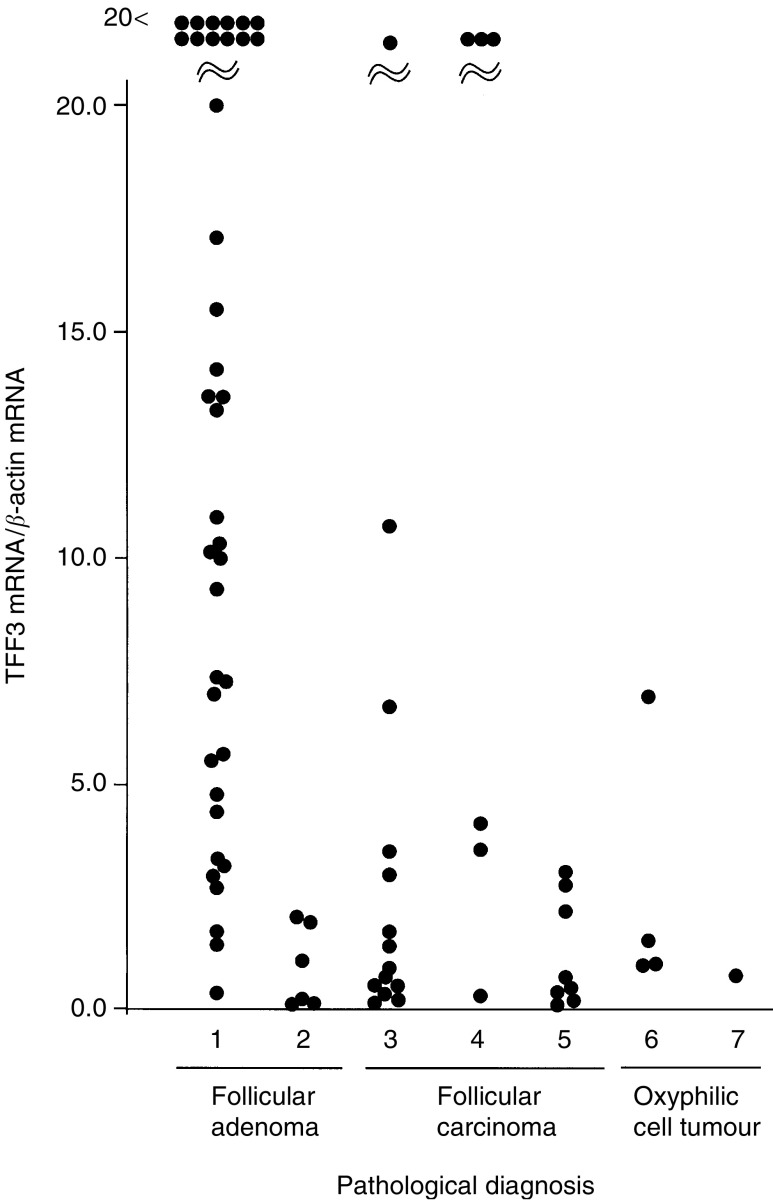
Expression levels of TFF3 mRNA relative to *β*-actin mRNA in follicular tumours. The results are shown as the mean from duplicate determinations. (1) Adenomas; (2) questionable adenomas; (3) minimally invasive carcinomas; (4) questionable minimally invasive carcinomas; (5) definite carcinomas; (6) oxyphilic cell adenomas; and (7) an oxyphilic cell carcinoma.

A total of 48 follicular adenomas and 29 follicular carcinomas were analysed. Five cases were oxyphilic cell tumours and eight cases were ‘definite’ carcinomas, which included seven widely invasive carcinomas and one minimally invasive carcinoma with lung metastasis. Among the other cases, six adenomas and six carcinomas were difficult to classify by the pathologist because of questionable pathological features, for example, insufficient penetration into the capsule. In follicular tumours, a significant difference in the expression levels of TFF3 mRNA was observed between follicular adenomas and carcinomas (Figure 3). Five oxyphilic tumours (four adenomas and one carcinoma) showed decreased expression levels of TFF3 mRNA. All eight definite carcinomas showed greatly decreased expression levels of TFF3 mRNA. All six questionable adenomas and three of the six questionable carcinomas showed decreased and greatly increased expression levels of TFF3 mRNA, respectively (Figure 4

).

**Figure 5:**
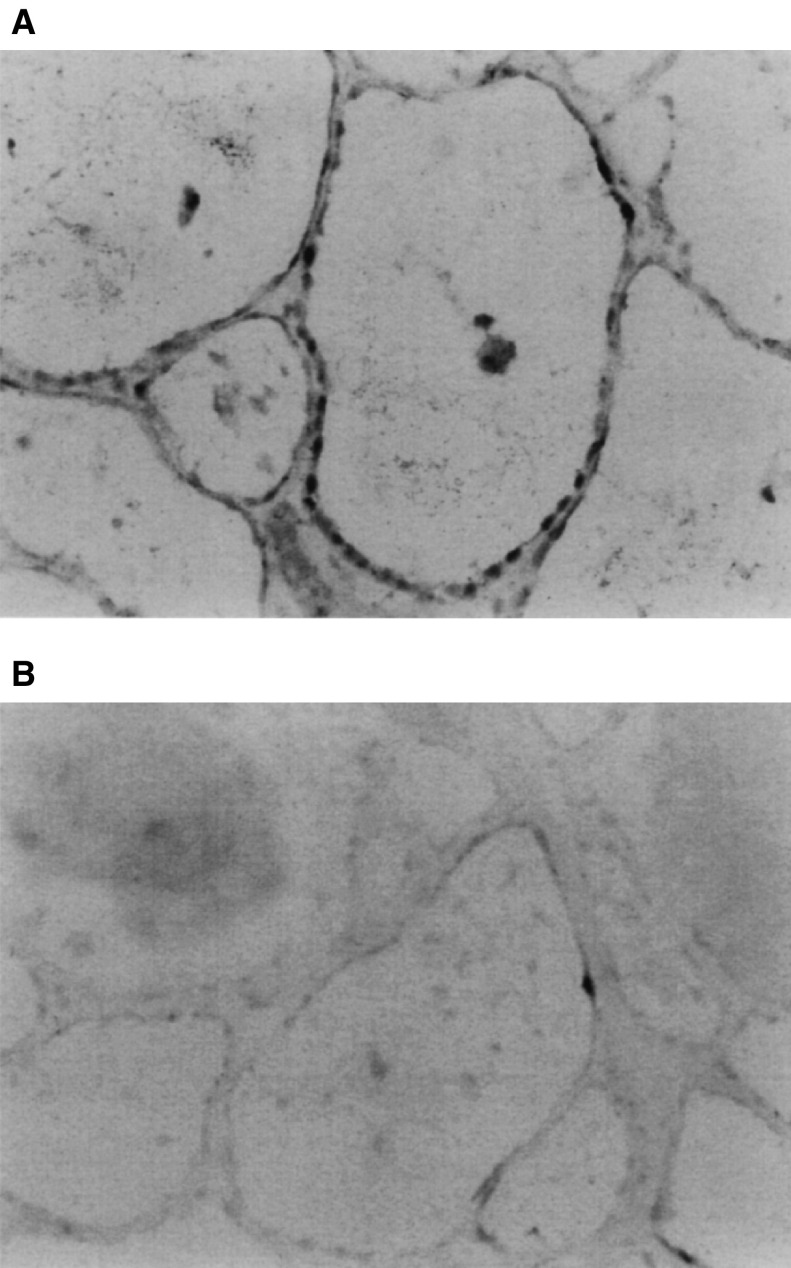
*In situ* hybridisation study using an antisense probe for TFF3 mRNA (× 100). (**A**) A follicular adenoma with abundant expression of TFF mRNA; and (**B**) a section from the same tumour stained with a sense probe as a negative control.

An *in situ* hybridisation study was performed to confirm that the expression of TFF3 mRNA was restricted to thyroid follicular cells. Strong staining of TFF mRNA in thyroid follicular cells was observed, which indicated that the majority of TFF3 mRNA is expressed by thyroid follicular cells but not stroma or blood cells (Figure 5

).

## DISCUSSION

Since SAGE analysis does not require special equipment or materials, it is more convenient and less expensive when compared with DNA chips or arrays. However, one of the limitations of the original protocol of SAGE is that the information on the gene expression profiles consists of occurrences of 14-bp sequences. This is a severe limitation, especially in the rapid second screening, because PCR primers are hard to design. In HDSS, the modified SAGE analysis constructs the gene expression profiles with 18-bp tags, with which we can easily proceed to the second screening by real-time quantitative RT–PCR. In fact, 37 of the 40 primers designed in this study amplified the corresponding cDNA successfully. However, in real-time RT–PCR analysis, 11 of the 37 primers set in this HDSS study showed no or discrepant differences between the two tissues used in the SAGE analysis. This was probably due to either: (1) the primer sets amplify different cDNA from that detected in the SAGE analysis, or (2) real-time RT–PCR is less sensitive than SAGE in the detection of the different expression levels of each gene, thus, differences between those genes whose expression differs slightly can be detected by SAGE but not by real-time RT–PCR. Among the 26 primer sets that proceeded to the second screening, only a primer set to amplify TFF3 cDNA showed a clear difference between adenomas and carcinomas. It is not likely that this fact indicates the limitation of HDSS as a tool for the rapid differential screening of mRNA. Rather, considering the fact that no mRNAs that distinguish follicular adenoma and carcinoma have been reported, there is probably a rarity of differentially expressed genes between follicular adenomas and carcinomas.

Using HDSS, we detected TFF3 as a differentially expressed gene between follicular adenomas and carcinomas. In a recent study using DNA arrays, Huang *et al* (2001) described the differential expression of TFF3 between normal thyroid tissues and papillary carcinomas. The trefoil factor family, which includes TFF3, is a relatively new family of peptides that bears the three-loop trefoil domain (Wong *et al*, 1999). They are mainly synthesised and secreted by mucin-secreting epithelial cells lining the gastrointestinal tract and have a close association with mucins. They are highly conserved during evolution and are resistant to heat, acid and enzymes. The TFF peptide action is still unknown; however, their abundant expression in various ulcerative conditions suggests an important role in mucosal defence and repair. Even less is known about TFF3, since it was the last to be identified. TFF3 is expressed mainly in the goblet cells of the small and large intestine and has recently been found abundantly in salivary glands (Devine *et al*, 2000). Some studies showed that immunoreactive TFF3 expression has been detected in neoplastic human colonic mucosa, colocalising with neutral mucin production, but loss of TFF expression is associated with tumour necrosis and advanced Dukes' stage (Machado *et al*, 1996).

The reason for the differential expression of TFF3 in follicular adenomas and carcinomas is not clear. Normal thyroids, adenomatous goitres and medullary carcinomas overexpress TFF3 mRNA. As these tissues are known to produce hormones, such as T3, T4 and calcitonin, the TFF3 peptide might play some roles in hormone secretion in the thyroid; thus, TFF3 mRNA can be regarded as a marker of cell differentiation.

Pathological diagnosis of follicular carcinoma is quite difficult and it usually requires a demonstration of vascular or full-thickness capsular invasion in a surgical specimen. In fact, in many cases, discrepant diagnoses are made by different pathologists. Further, by classification of WHO, which does not refer to the biological characteristics of the tumour cells but only to the minute sign of invasion, it is not surprising that a considerable number of ‘biological’ follicular carcinomas are diagnosed as ‘pathological’ follicular adenomas. However, to what extent this has happened is quite difficult to estimate because evident malignant features of follicular carcinoma such as distant metastasis often appear many years after surgery. Thus, some kind of objective molecular criteria that distinguish benign and malignant follicular tumours, which reflect the biological characteristics of the tumour cells, are necessary for making more correct diagnoses. Differentially expressed mRNAs between follicular adenomas and carcinomas can be a good indicator in such molecular-based classification.

The expression of TFF3 mRNA is decreased significantly in follicular carcinomas compared with follicular adenomas. Eight definite carcinomas with wide invasion or distant metastasis showed greatly decreased expression. These results indicate that the decreased expression of TFF3 is closely related to the malignant features of follicular cells. Interestingly, as shown in Figure 4, the majority of follicular adenomas with low expression levels of TFF3 showed questionable pathological features. On the other hand, three of the four follicular carcinomas with high expression levels of TFF3 were pathologically questionable. These results strongly suggest the possibility that TFF3 mRNA expression is an objective indicator of malignancy in follicular tumours and may be useful in diagnosing follicular tumours, which are difficult to classify by pathological means. However, it is not possible to determine to what extent this hypothesis is correct, and long-term observation of the two groups, one with high and the other with low expression of TFF3 mRNA, is needed before we can clarify the usefulness of these molecular-based criteria. In five oxyphilic cell tumours, regardless of pathological classification, low expression levels of TFF3 mRNA were observed. Although analysis of a large number of samples is needed, TFF3 mRNA expression may not be useful for determining the malignant features of oxyphilic cell tumours.

In our preliminary study, differentiation of follicular adenomas from follicular carcinomas was not possible by the *in situ* hybridisation study since both tumour cells showed a positive staining of TFF3 mRNA (data not shown). These results were not surprising, however, since even in widely invasive follicular carcinomas, TFF3 mRNA was expressed at almost the same level as *β*-actin mRNA. This fact suggests that the use of TFF3 mRNA or peptide in the diagnosis of follicular tumours by morphological methods, such as *in situ* hybridisation or immunohistochemistry, might be difficult, although in some cases, modifications of the methods to reduce the sensitivity might work.

Several trials have been performed to diagnose follicular carcinomas preoperatively, but unfortunately most of them were not satisfactory for clinical use. A recent study showed the usefulness of immunohistochemical study of galectin-3 for the diagnosis of thyroid follicular carcinoma (Bartolazzi *et al*, 2001). However, in another study, no significant differences were observed between follicular carcinomas and adenomas by quantitative measurement of galectin-3 mRNA (Bernet *et al*, 2002). Zeiger *et al*. measured the expression levels of telomerase reverse transcriptase (hTERT) in follicular tumours and showed that this method was to some extent useful in diagnosing follicular carcinomas, although they also described interference due to contamination by lymphocytes, which expressed a considerable copy number of hTERT mRNA (Zeiger *et al*, 1999). Kroll *et al* reported some promising data in which PAX8-PPAR*γ*1 fusion mRNA and protein were detected in 60% of thyroid follicular carcinomas but not in follicular adenomas, papillary carcinomas, or multinodular hyperplasias. However, because their study employed only a small number of follicular carcinomas, additional examination is necessary (Kroll *et al*, 2000). As TFF3 mRNA is abundantly expressed in thyroid tumours, measurement of the copy number in FNAB samples may not be difficult. Thus, when an accurate system to measure the quantity of TFF3 mRNA is established, it may be possible to distinguish follicular adenomas and carcinomas preoperatively.
